# The Herts and Minds study: feasibility of a randomised controlled trial of Mentalization-Based Treatment versus usual care to support the wellbeing of children in foster care

**DOI:** 10.1186/s12888-019-2196-2

**Published:** 2019-07-10

**Authors:** Nick Midgley, Sarah Jane Besser, Pasco Fearon, Solange Wyatt, Sarah Byford, David Wellsted

**Affiliations:** 1 Child Attachment and Psychological Therapies Research Unit (ChAPTRe), UCL / The Anna Freud National Centre for Children and Families, London, UK; 2 The Centre for Health Services and Clinical Research, The University of Hertfordshire, Hatfield, UK; 3 Research Department of Clinical, Educational and Health Psychology, University College London, London, UK; 4 Institute of Psychiatry, Psychology and Neuroscience, King’s College London (KCL), London, UK

**Keywords:** Foster care, Mentalization, Mentalization-based treatment, Feasibility study, Looked after children, Randomised controlled trial

## Abstract

**Background:**

There is a lack of well-designed randomized controlled trials (RCTs) to investigate the efficacy of psychological therapies for children in foster care with emotional and behavioural difficulties. Mentalization-based therapy (MBT) focuses on supporting the carer-child relationship by promoting reflective capacity. This study examined the feasibility and acceptability of an RCT of MBT, delivered in a family-format, for children who are in foster care in the UK.

**Method:**

Herts and Minds was a phase II, blinded feasibility RCT with follow-up of at 12 and 24 weeks post-randomisation. Participants were children (age 5–16) in foster care referred to a targeted mental health service, who had some level of difficulty as identified by the Strengths and Difficulties Questionnaire (SDQ). Aims were to assess: the feasibility of recruitment processes and study uptake; capacity to train mental health practitioners to deliver MBT to an acceptable level of treatment integrity; establish acceptability and credibility of MBT as an intervention for children in foster care; establish feasibility and acceptability to participants of conducting an RCT; and estimate the likely treatment efficacy effect size. Participants were randomly allocated to either MBT (*n* = 15) or Usual Clinical Care (UCC) (*n* = 21) individually or in sibling groups. A range of qualitative and quantitative data was gathered to assess feasibility.

**Results:**

Feasibility was established with regard to: capacity to recruit participants to a study; capacity to train mental health practitioners to deliver MBT to an acceptable level of treatment integrity; acceptability and credibility of MBT; and feasibility and acceptability to participants of conducting an RCT. A number of issues made it difficult to estimate a likely treatment efficacy effect size.

**Conclusion:**

With modifications, it is feasible to run an RCT of MBT for children in foster care. Both the therapy and research design were acceptable to participants, but modifications may be needed regarding both the timing of assessments and the identification of appropriate primary outcome measures. Given the lack of evidenced based therapies for this population, such a trial would be a significant contribution to the field. Findings may be useful for other groups planning clinical trials of psychological therapies for children in foster care.

**Trial registration:**

ISRCTN 90349442. The trial was retrospectively registered on 6 May 2016.

**Electronic supplementary material:**

The online version of this article (10.1186/s12888-019-2196-2) contains supplementary material, which is available to authorized users.

## Background

Children who are taken into the social care system are referred to in the UK as ‘looked after children’ and are an extremely vulnerable group who are at high risk of experiencing poor physical [1] and mental health [2] and long-term maladjustment [3, 4]. Between 2010 and 2017 the number of children who are in care in England has risen by 10%, with the largest proportion (74%) living in foster care [2]. The majority of these children were taken into care as a consequence of abuse, neglect and/or family dysfunction [2]. Estimates of the proportion of looked after children with mental health difficulties are high, ranging from 45% [5] to 72% [6].

Mentalization-based Treatment (MBT) is a relatively new approach to psychological therapy, which has established an evidence base for adults with borderline personality disorder [7, 8] and adolescents who self-harm [9]. ‘Mentalization’ refers to the capacity to make sense of each other and ourselves, implicitly and explicitly, in terms of subjective states and mental processes. Greater mentalizing capacity in parents and carers is associated with improved outcomes for children with emotional and behavioural difficulties [10]. Although MBT has yet to be evaluated systematically in work with children in foster care, the approach has been manualized, and includes many of the features set out in the UK guidelines [11] as key elements of best practice for work with looked after children.

Researchers have identified a number of challenges to conducting high-quality research with looked after children in the social care setting [12]. These include the complexity of defining emotional health and well-being of looked after children, and the heterogeneity among them; the lack of measures appropriately validated for use with this particular population; the lack of training in research methodology among social work professionals, creating a culture in which ‘practice-based wisdom’ is valued over evidence based practice [12, 13]; and practical difficulties in accessing participants and gaining consent for participation in research [14]. For these reasons there is a lack of well-designed studies and randomized controlled trials to investigate the efficacy of psychological therapies for looked after children [15]. The overall aim of this study was therefore to establish the feasibility of conducting a clinical trial of MBT for children in foster care with emotional and behavioural difficulties.

## Methods

### Trial design

The study was a parallel group, single centre, feasibility randomised trial with two arms, conducted over a 2 year period. Details of its design can be found in the study protocol [16, 17].

The objectives were to: (1) assess the feasibility of recruitment processes and study uptake; (2) test capacity to train mental health practitioners to deliver MBT to an acceptable level of treatment integrity; (3) establish acceptability and credibility of MBT as a treatment intervention for looked after children; (4) to establish the feasibility and acceptability to participants of conducting a randomised clinical trial; (5) to establish the feasibility and acceptability to participants of collecting resource use and health-related quality of life data to support economic evaluation; and (6) to estimate the likely treatment efficacy effect size.

The study was conducted in a Child and Adolescent Mental Health Services (CAMHS) Targeted team within a single NHS Trust. Unlike the generic CAMHS teams in the same NHS Trust, the Targeted CAMHS team was set up specifically to work with children in care or on the edge of care (i.e. at risk of being removed from their families by social services), because thresholds for generic CAMHS teams often meant that children in care were not offered a service. It was also considered important that the Targeted CAMHS team, which was made up of Clinical Psychologists, Social Workers and Play Therapists, had specific expertise in working with children in care and foster carers, as this context could shape their mental health support needs. The Targeted CAMHS team did not include a Child Psychiatrist, so those children needing specific psychiatric input (e.g. an assessment for autism or ADHD, or where risk of self-harm was significant) were referred to the generic CAMHS team.

The baseline research visit was conducted before any treatment was initiated. Participants were randomised to either MBT or Usual Clinical Care (UCC) as soon as possible after the baseline assessment. Participants were then followed-up at 12 and 24 weeks post-randomisation.

### Participants selection

The target population was children in foster care referred to the Targeted CAMHS team, aged between 5 and 16, who had been with their current foster carer for at least 4 weeks, and who had a Strengths and Difficulties Questionnaire (SDQ) score that indicated some level of difficulty (≥13). Children and their foster carers were included in the study if, following an initial consultation with the Targeted CAMHS team, they were considered to be a suitable referral for the Service (e.g. the child was about to move to a new placement in a different area). Participants were excluded if they were signposted to another service, e.g. an emergency/crisis referral requiring psychiatric assessment, or if they were in need of a different treatment (e.g. an educational psychology assessment) within or outside of CAMHS.

### Procedure

The recruitment process is fully outlined in the trial protocol [15, 16]. The consent process was complex, since the process of consent varied depending on the legal status of the child. Prior to obtaining consent from the foster carer and child, consent was required from the birth parent and, or the local authority representative. For children on full or interim care orders, consent was obtained from a representative of the Local Authority; for children on voluntary placements, consent was sought from at least one of the child’s birth parents, depending on which parent was involved in the child’s care.

Children were individually randomly assigned to either one of the two treatment groups, unless they were part of a sibling group, in which case they were randomised as a block. Randomisation was also stratified by age (5–11 and 12–16), and sex. While allocation was not concealed from either the children/young people or the foster carers, the researchers conducting assessments and the trial statistician were blind to the trial arm allocation. The trial manager was not blind to trial arm and had the role of allocating children to the correct trial arm following randomisation.

### Interventions

#### Mentalization-based treatment

MBT is a short-term manualized treatment, offering up to 12 weekly sessions, and delivered in a family format by existing clinicians working in the Targeted CAMHS team. The approach includes a combination of psycho-education about attachment and mentalizing in children with histories of maltreatment; consultations with the professional network around the child, when required; and direct relational work, tailored to the needs of each foster family, aimed at helping foster families understand their foster child’s needs and feelings, encouraging sensitive parenting and tackling problematic patterns of foster family interaction. This manualized adaptation of MBT pays particular attention to promoting mentalizing in the foster carer and developing reflective practice for all professionals working with the referred child.

#### Usual clinical care (UCC)

Participants in the usual care arm were offered up to 12 weekly sessions of therapy by the Targeted Team. Clinicians employed by the Targeted CAMHS team have varied training, including social work and clinical psychology. Decisions for what therapy to use for each child as part of usual care were made on the basis of the service’s usual practice, which was based on the ‘Choice and Partnership Approach’ (CAPA: [17]). Usual care consisted of a mix of other therapeutic techniques, including cognitive behavioural therapy, play therapy and theraplay.

### Data collection

A range of qualitative and quantitative data was collected, in order to address the range of feasibility aims set out above. Full details of the measures and semi-structured interview schedules used have been described in the protocol paper [16]. Table 1 provides details of each of the feasibility questions, and the associated outcomes measures and methods of assessment. All assessments were administered to participants in both trial arms.

**Table 1:** Feasibility questions, assessments and assessment methods

Feasibility Question	How was feasibility assessed?	Assessment methods	When was feasibility assessed?
Is it feasible to recruit participants?	Number of families and children referred to the Targeted CAMHS team, documenting reasons for ineligibility or study decline	Recruitment log	From trial open to close.
It is possible to train therapists to an appropriate level of treatment integrity?	Skill level in delivering MBT was assessed in the MBT and UCC study arms Therapists’ views regarding treatment integrity	MBT-Fostering- Adherence and Competence Scale [MBT-F-ACS; (Wood S, Besser S, Midgley N: MBT-Fostering- Adherence and Competence Scale (MBTF-ACS), unpublished)] Focus groups with targeted team clinicians	During and after intervention period
Is MBT acceptable and credible?	Monitoring of attendance and drop out of therapy sessions Participants’ and professionals’ views regarding treatment acceptability	Treatment attendance log Semi-structured interviews / focus groups with foster carers, social workers and targeted team clinicians	Intervention period Interviews at final follow-up (24 weeks)
Is a trial feasible and acceptable?	The extent to which children, carers, and therapists complete study assessments Participants’ views of the study procedures, and facilitators and barriers to participating in the study Withdrawal from treatment, and/or from the study	Completion rate of all measures (see Additional file 1) Semi-structured interviews with foster carers Recruitment log	Final analysis at trial close Interviews at final follow-up (24 weeks) Throughout the trial
Is it feasible to collect resource use and quality of life data for economic evaluation?	Completion of a) resource use schedule modified for the population of interest and b) health-related quality-of-life assessment tools	Child and Adolescent Service Use Schedule (CA-SUS) [18] and Child Health Utility (CHU-9D) measure of health-related quality of life [19]	Baseline, weeks 12 and 24
What is the likely effect size?	The likely effect size for the MBT intervention, compared to UCC	Strengths and Difficulties Questionnaire (SDQ, Goodman and Goodman 2012) [20]	Baseline, weeks 12 and 24

Note: See Additional file 1 for full details of all measures

### Data analysis

The purpose of the statistical and qualitative analysis was to evaluate the feasibility of undertaking a full clinical trial. The quantitative analyses relating to trial feasibility was mainly descriptive, estimating means and standard deviations, and differences between means with confidence intervals. Mean and standard deviations were estimated for continuous measures, and proportions for events (proportion of patients randomised). Differences between group means were estimated as a mean difference with a confidence interval. Adjusted group differences were estimated using a hierarchical regression model and were adjusted for baseline values of covariates. In estimating proportions, the denominator was determined from all young people referred.

The acceptability and credibility of MBT as a therapy in the context of a targeted team was examined both quantitatively, by looking at overall levels of attendance and drop-out from treatment; and qualitatively, drawing on data from the semi-structured interviews and focus groups. Quantitative data analysis using descriptive statistics was carried out using the STATA statistical package. The feasibility and acceptability of collecting resource use and health-related quality of life data suitable for economic evaluation were examined in terms of completion rates and the service use measure was adapted for clarity and comprehensiveness following feedback from participants.

Semi-structured interviews of participants in the MBT arm and focus group data were analysed using thematic analysis [21] in Nvivo version 11. The thematic analysis yielded a list of broad themes, which were then arranged by each of the study research questions, so that themes relevant to each question could be used to answer it. Information coded under these themes were then used to answer the question about the feasibility of this aspect of the research methods. For the semi-structured interviews, a check for credibility of the thematic analysis was conducted, whereby 10% of transcripts were reviewed by one of the trial team research assistants. Overall there was high agreement on the main themes that were identified. Any disagreements were discussed, by going back to the transcripts and considering different understandings of the data, until consensus was reached.

## Results

Each of the 6 research aims of this feasibility study are addressed below. For the reporting of the qualitative analysis the following terms are used to give an approximate idea of how many participants may have expressed particular views: a few (less than 20%); some (20–50%); many (51–80%); most (over 80%); all (100%).

### Aim 1: to assess the feasibility of recruitment processes and uptake to the study

**Fig. 1:**
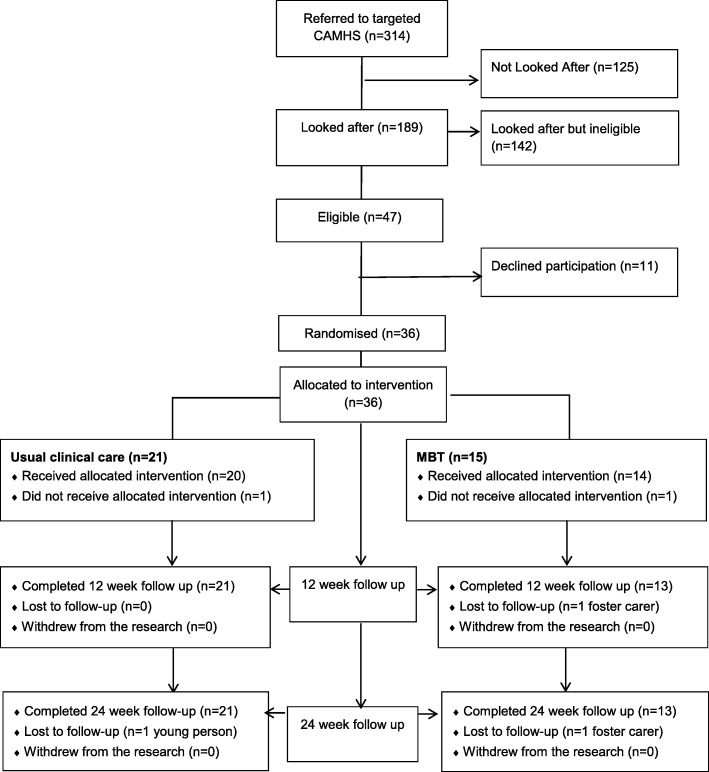
Flowchart from referral to final research follow-up

As illustrated in Fig. 1, 314 young people were referred into the Service over a period from April 2016 to July 2017. Of these, 189 were looked after children, whilst the others were children on the edge of care. Of the 189 looked after children, 47 were eligible to participate in the study. Reasons for non-eligibility are shown in Table 2.

**Table 2:** Reasons for non-eligibility

Reason	N
Child referred to other service	55
SDQ score < 13	30
Age < 5	14
Child not placed with foster carer	13
No funding in place for treatment	10
Age > 16	7
Inappropriate referral	5
Missing referral data at study close	4
Young person moving out of county	3
Child unable to understand questionnaires	1

Of the 47 eligible children and young people, 36 (77%) were recruited and enrolled to the study and 33 families (92%) completed the final follow up sufficient for analysis. Among the 11 children who were eligible but did not participate, the source of refusal was either the foster carer (5), the young person (1) or the local authority (5). Reasons given mostly focused on feeling that there was ‘too much going on’ for the child, or else that it was ‘not the right time’ to be part of a study.

Owing to the method of random allocation to treatment arms, 21 children were randomised to the Usual Clinical Care arm, and 15 to the MBT arm of the study. The characteristics of the children recruited to the study are shown in Table 3. Children in the MBT arm were slightly older than the UCC arm, were more likely to be on a full care order, had been in care longer and had more previous foster care placements.

**Table 3:** Child characteristics according to group allocation

Young People	N	All	n	Usual Care	n	MBT
Age (mean, sd, years)	36	10.6 (2.7)	21	10.2 (3.0)	15	11.1 (2.2)
Sex (male %)	36	20 (56%)	21	12 (57%)	15	8 (53%)
Ethnicity (White British %)	36	32 (89%)	21	18 (86%)	15	14 (93%)
Time in foster care (mean, sd, years)	36	2.4 (2.5)	21	1.9 (2.3)	15	3.1 (2.7)
Siblings (yes %)	36	35 (97%)	21	21 (100%)	15	14 (93%)
Placed with siblings (yes %)	34	14 (41%)	20	9 (45%)	14	5 (36%)
First in care (age, mean, sd)	33	4.8 (3.3)	19	5.2 (3.3)	14	4.4 (3.3)
Previous placements (median, min/max)	32	1 (0/10)	19	1 (0–10)	13	2 (0–7)
Type of care order						
Full		30 (83%)		16 (76%)		14 (93%)
Interim		5 (14%)		4 (19%)		1 (7%)
Voluntary		1 (3%)		1 (5%)		0 (0%)

From the analysis of the semi-structured interviews carried out at the end of the study, foster carers reported that they were generally satisfied with the method of recruitment, which involved them receiving the first contact about the research from their own or the child’s social worker, to check with them that they were happy for a member of the research team to be in touch to explain more about the study. Some said it gave them confidence in the study to know that their social workers were familiar with it. No issues with the consenting process were raised in the interviews with foster carers and children.

### Aim 2: to test capacity to train mental health practitioners to an acceptable level of treatment integrity

Therapist training was assessed from skill level scores of the MBT-F-ACS, where a higher score shows a greater level of skill. Based on the availability of data, and in proportion to the number of cases seen by each therapist, 11 usual care and 13 MBT sessions were rated. Table 4 sets out the mean MBT-ACS scores across the two treatment arms, by session and also by therapist.

**Table 4:** Average MBT-F-ACS skill level scores of therapists by trial arm

	Usual Care					MBT				
	N	Mean	IQR	Rating ≥ 24	Rating ≥ 32	N	Mean	IQR	Rating ≥ 24	Rating ≥ 32
All sessions	11	22.5	10	6/11	0/11	13	39.5	6	13/13	11/13
By therapist	4	22.5	10	3/4	0/4	3	39.5	6	3/3	3/3

Note: A score of 24 is equivalent to an average of 3 per item, where 3 indicates an ‘acceptable’ level, and a score of 32 is equivalent to a score of 4 per item, where 4 indicates an ‘adequate’ level

In line with the original guidance on the use of the MBT-ACS for use with adults [22] cut-off score of 24 or above was pre-determined as indicating adherence to the MBT model (indicating an ‘acceptable’ level of adherence). All 13 MBT sessions were rated as adherent; with 6/11 usual care sessions also meeting levels of adherence to an MBT approach. Some more recent studies using the MBT-ACS have set a higher cut-off score of 32 for assessing treatment fidelity. Table 4 shows that when using a cut-off score of 32 or above, sessions in the MBT arm met the criteria in 11/13 sessions, whereas no sessions in the usual care arm met the criteria.

In the focus group with therapists at the end of the study, all the therapists who were in the MBT arm of the study indicated that supervision was highly important to maintaining treatment fidelity. They said that supervision was key to being able to deliver the therapy and ‘stay on model.’ They were all agreed that if they had only attended the 4-day training, and not had the on-going supervision, it would have been very difficult to remain adherent to the MBT approach.

Therapists in both arms of the study believed that aspects of the MBT model were inherent to how all therapists would usually work, whether they had been specifically trained in MBT or not. This viewpoint is consistent with the findings from the MBT-ACS, which also indicated that usual care therapists were using a certain amount of techniques associated with a mentalizing approach, even if MBT were using a greater amount of these techniques overall.

### Aim 3: the acceptability and credibility of MBT as a treatment intervention for children in foster care referred to a targeted CAMHS team

There was a very high level of treatment attendance in both trial arms (90% in MBT vs 92% in UCC). There were also no drop-outs from either of the trial arms throughout the study, although three participants in the MBT arm (a sibling group) were re-assessed as not needing an intervention after one session, following a change in placement. Safety reporting was robust, with no serious adverse events attributed to the therapy intervention. In both arms, the number of reported events (e.g. change of school, change of social worker, changes in contact arrangements, placement breakdown, involvement with youth justice system or school exclusions) reduced by more than two thirds during the study relative to baseline.

In the semi-structured interviews, most foster carers and children reported a very positive experience of the MBT sessions. The MBT therapists were described by foster carers and children as approachable, friendly, and able to make them feel comfortable. Many helpful elements of the session were reported, such as the possibility for the child to play during the session while talking. Foster carers spoke about how they felt supported to develop strategies to help the children in their care, such as using emotion cards to gain understanding of emotions or writing letters to their foster child when they were going out and would be away for a long period of time, to help the foster child to understand that they would be coming back.

Out of the twelve foster carers who were interviewed, two reported less positive experiences, with the focus especially on the delays in receiving therapy from CAMHS, which they both found highly concerning for the children in their care. Of these two, once therapy had begun, one spoke of the MBT therapy itself as a positive experience, but the other one did not, as she felt ‘criticised’ by her therapist, and felt that she was being told that she was parenting incorrectly.

Among the CAMHS therapists who delivered MBT, there were many positive comments to suggest that they found the intervention acceptable and appropriate for this particular client group, and distinct from usual care. In particular most found the focus on developing joint curiosity, rather than ‘coming up with solutions’ (i.e. feeling that their role was to suggest behavioural strategies for managing a child’s difficulties), a key shift from their usual practice. They felt that a mentalizing approach was useful for the children, and believed it was valuable for children to hear a conversation between the foster carer and therapist where they were both being curious in trying to understand the young person.

### Aim 4: to establish the feasibility and acceptability of conducting a randomised controlled trial

Retention of participants in the study was very high, with no withdrawals from the study at any point from baseline through to the final research assessment at 24 weeks, even if there had been a change of foster carer during the course of the study. Although one foster carer was lost to follow-up, data were obtained from her foster child; and one foster child was lost to follow-up, but data were collected from her foster carer. All young people had engaged in some therapy by 24 weeks, however the rate at which children and carers engaged in therapy was much slower than had been expected; by 12 weeks (which was the anticipated end of treatment assessment point), no children had completed therapy (other than the sibling group who had been re-assessed as not needing therapy after one session), and the median number of sessions attended by that point was three (in both trial arms). By the trial end (24 weeks) only 38% of patients in usual care had completed therapy, and 27% in the MBT arm, with the median number of sessions attended being 7 in UCC and 6 in MBT.

The proportion of participants who completed each questionnaire at each time point was very high (≥87%) apart from a Goal Based Outcome measure (less than 50%), and the teacher-reported SDQ (less than 40%). The reasons for the lower completion rates on these measures were related to difficulties with the initial goal-setting process in therapy (GBOM) and the challenges of contacting school staff (teacher-rated SDQ). The blinding of the research assistants and statisticians was maintained throughout the trial.

Analysis of the semi-structured interview data with foster carers showed that the research design and assessments were mostly perceived very positively and were seen as acceptable to participants. Aspects that they felt had worked well in terms of the research assessments were: the flexibility and friendly nature of the research team, location of the research visits (at foster carer homes), and relatively short duration of the research visits (ranging from 30 min to 2 h, baseline visits were usually longer than follow-ups). Having two researchers present was seen to be helpful when the participants were part of a sibling group. Some foster carers and children also opted to complete the measures in their own time, rather than during the researcher’s visit. The research team offered to read the questionnaires to children, and for very young children this was essential.

All foster carers said they would recommend taking part in the study to other carers, and some children said they wanted to continue with the research and enjoyed completing the questionnaires. A few children reported that they found the questions boring, but most said they were fun and they enjoyed completing them.

Many foster carers commented that they enjoyed the process of completing the questionnaires, because it gave them the opportunity to see the changes happening and reflect on how their child was progressing. Most foster carers also reported no problem with the randomisation process and stated that they did not have a preference for which group they were allocated to, as long as appropriate support was offered. Many said they would not have known the difference between the different types of therapy and had not thought about which group they were in during therapy.

### Aim 5: to establish the feasibility of collecting resource-use data, for the purpose of calculating relative cost-effectiveness

Completion of the economic evaluation measures (Child Health Utility 9D (CHU-9D) and Child and Adolescent Service Use Schedule (CA-SUS)) [18, 19] were high, exceeding 81% in all cases. Apart from the one participant who was lost to follow-up, a full data set was obtained. The high level of data completion indicated that both measures are appropriate, and the research team and participants did not report any difficulties with using these measures.

### Aim 6: to constrain a preliminary estimate of likely treatment efficacy effect size (treatment outcome measures)

Following Cocks and Torgerson (2013), treatment outcome data were gathered in order to evaluate whether the likely effect size for the MBT intervention, compared to UCC, was larger than 0, based on the primary outcome - carer-reported SDQ [23]. The lower and upper limits for the effect size (d) are estimated as a 90% confidence interval. Although the SDQ was the identified as the most likely primary outcome given its mandated use with looked after children in the UK, SDQ ratings from the young people, among other self-report scales (see Additional file 1) were also evaluated. Differences between the MBT and usual care groups were examined using a mixed model (clustered within young person), adjusted at baseline for SDQ score at referral and for Foster Carer Reflective Functioning, as assessed by the Five Minute Speech Sample [24].

A different pattern of outcomes was observed for the carer-reported and self-report SDQ. For the carer-reported SDQ the usual care group reported an improvement over time (− 2.7) which was not reported in the MBT group (+ 0.5). The observed improvement for the usual care group was larger in the Internalising score (− 1.5) than in the Externalising score (− 1.2). The equivalent changes in the MBT group were marginal (Internalising + 0.7, Externalising − 0.2). The associated effect size was > 0 at the lower bound for the 90% confidence interval for the Scale Score at 12 weeks (d’ = − 0.44 [− 0.8, 0.0]), and for the Internalising score at 12 and 24 weeks (d’ = − 0.35 (− 0.7, 0.0), d’ = − 0.48 [− 0.8, − 0.1]).

For the young person’s self-reported SDQ scores a different pattern was observed (Table 5). Overall the scores reported by the young people were lower than their carers by 4 to 5 points. In addition, the young people in the MBT group reported an improvement over time (− 1.3) but little change in the usual care group (+ 0.3). The observed changes in the Internalising scores (− 2.7 MBT, + 1.0 UCC) were reversed for the Externalising score (+ 1.4 MBT, − 0.6 UCC). However the adjusted effect size shows a significant advantage for the MBT group at 12 and 24 weeks (d’ = 0.7 [0.2, 1.1] and 0.8 [0.3, 1.2]), which is observed for Internalising (d’ = 1.0 [0.5,1.5] and d’ = 1.3 [0.7,1.7]), but not Externalising d’ = 0.1 [− 0.3,0.6] and d’ = 0.1 [− 0.3,0.6]). Notably the effect size is consistently, and considerably larger than 0, with an advantage for the MBT group for the Scale Score, apparently driven by the Internalising subscale. What is particularly noticeable is that the effect size advantage for MBT for the scale and Internalising scores are consistently and noticeably larger in young people than the advantage for UCC reported by Foster Carers..

**Table 5:** SDQ scores for Foster Carer and Young Person reports at each time point

		UCC		MBT		Adjusted difference	
Scale Score	Total	N	Mean (sd)	N	Mean (sd)	90% CI	d’ (90% CI)
Total SDQ score (foster-carer report)							
Baseline	36	21	19.8 (6.9)	15	18.5 (7.1)	–	–
12 Weeks	35	21	18.9 (4.6)	14	19.1 (6.6)	−1.7 (−5.8, 2.4)	−0.31 (−0.7,0.1)
24 Weeks	35	21	17.1 (7.0)	14	19.0 (7.4)	−3.1 (−8.2, 1.9)	−0.44 (− 0.8,0.0)
Internalising sub-scale (foster carer report)							
Baseline	36	21	7.9 (4.5)	15	6.7 (4.3)		
12 Weeks	35	21	7.4 (3.3)	14	7.3 (4.1)	−1.3 (−3.9, 1.4)	−0.35 (−0.7, 0.0)
24 Weeks	35	21	6.4 (4.2)	14	7.4 (4.6)	−2.1 (−4.9, 0.7)	−0.48 (− 0.8, − 0.1)
Externalising sub-scale (foster carer report)							
Baseline	36	21	11.9 (4.5)	15	11.8 (3.9)		
12 Weeks	35	21	11.5 (4.0)	14	11.9 (3.9)	−0.2 (− 2.5, 2.2)	− 0.04 (− 0.4, 0.3)
24 Weeks	35	21	10.7 (4.2)	14	11.6 (4.0)	−0.8 (−3.5, 1.9)	− 0.20 (− 0.5, 0.2)
Total SDQ score (young person self-report)							
Baseline	18	9	12.2 (8.0)	9	14.2 (5.9)		
12 Weeks	20	11	13.0 (7.7)	9	12.8 (6.9)	4.9 (−1.0, 10.8)	**0.67 (0.2, 1.1)**
24 Weeks	20	11	12.5 (6.2)	9	12.9 (4.8)	4.2 (−0.8, 9.3)	**0.76 (0.3, 1.2)**
Internalising sub-scale (young person self-report)							
Baseline	18	9	4.2 (4.5)	9	6.3 (3.9)		
12 Weeks	20	11	5.4 (4.5)	9	4.9 (4.1)	4.5 (0.8, 8.2)	**1.04 (0.5, 1.5)**
24 Weeks	20	11	5.2 (3.4)	9	3.6 (2.7)	4.0 (0.4, 7.6)	**1.30 (0.7, 1.7)**
Externalising sub-scale (young person self-report)							
Baseline	18	9	8.0 (4.6)	9	7.9 (3.4)		
12 Weeks	20	11	7.7 (3.7)	9	7.9 (4.4)	0.6 (−2.0, 3.2)	0.15 (−0.3, 0.6)
24 Weeks	20	11	7.4 (3.6)	9	9.3 (5.0)	0.4 (− 2.2, 3.0)	0.09 (− 0.3, 0.7)

*Note: The observed scores at each time point are reported with the standard deviation. Adjusted difference between groups was estimated using a hierarchical regression model, with adjustment for baseline SDQ and Foster Carer Reflective Function*

The figures in bold indicate effect sizes (d) where the confidence interval for d does not include zero, indicating confidence (95%) that the effect size is >0

In the semi-structured interviews, foster carers reported a variety of improvements post-intervention, such as helping normalise the child’s problematic thoughts and behaviours, reduction of negative outbursts and aggression, more stable mood and the young person being more comfortable in the foster care placement. In line with the theoretical model of MBT, changes noted by many foster carers (and some children) related to their own ability to see things from the other person’s perspective:

*‘I mean, I knew he was troubled, but I didn't really realise the extent to how much, how troubled he was. So I think it's given me a much bigger insight into what's going on in [child’s name]'s head, and his feelings about the past, and how it's affecting his day-to-day life now.’* (Foster Carer of 10 year old boy)

Many children said they thought the therapy was helpful, for example one said:

*‘to let my feelings out… it’s helpful that I can tell them my worries and then I won’t have…any more worries*.’ (Young Person, female, aged 9)

However, three foster carers said it was difficult to ascertain what had brought about the changes in the child - they stated that changes could be attributed to maturation, and one suggested that acceptance from the young person that she needed help was key.

When asked directly if they found anything unhelpful about MBT most participants emphasised that their experience of therapy had generally been positive. All but one reported that they would definitely recommend the therapy to others. Some aspects of therapy that foster carers would like to see improved were issues to do with the location and timing of sessions rather than the content or style of therapy sessions. In particular they raised concern that the clinic location was run down and the timing of the therapy sessions clashed with the child or young person’s school hours.

## Discussion

This study provides evidence that it is possible to run a full scale RCT comparing MBT in a family format with usual clinical care for children in foster care within a Targeted CAMHS team setting. Prior to the present study, there was a genuine question of whether it is feasible to run a trial of this nature with a population of children in foster care. Despite the challenges identified in previous literature [12, 13, 25] the present trial largely achieved its aims. Overall there was evidence to suggest that a clinical trial is feasible in terms of (1) recruitment and retention of children and foster carers to both the therapy and the research, (2) training CAMHS clinicians to deliver MBT in a family format, (3) differentiation of the trial arms, (4) acceptability and credibility of MBT, (5) collection of a full data set, including resource use. The successful collection of resource use data indicates that it would be feasible to conduct a full health economic analysis in a full-scale trial.

As a feasibility study, the study was not powered to detect group differences in outcomes. Nevertheless, we note that even in this small sample, an indication of significant benefits were found for MBT compared to usual care for child-reported Internalizing problems. However, we also observed some marginal effects favouring usual care when reported by foster carers. We treat these findings with caution as the study was not designed to have power to detect treatment effects, nor achieve adequate balance across groups at baseline, and these effect estimates therefore are associated with considerable uncertainty. Nevertheless, the results highlight the potential for outcomes to be discrepant depending on reporter, which is a well-documented phenomenon in child mental health research [26]. A future trial would need to consider this issue when determining the optimal primary outcome measure.

There is also evidence that clinicians can be trained to deliver MBT in a family format to a good standard, based on a relatively brief (4 day) training. The treatment integrity data suggests that therapists in both trial arms used some elements of a mentalization-based approach, but MBT trained therapists scored consistently higher, demonstrating that training (together with regular supervision) did have an impact on practice, and that CAMHS therapists can be trained to deliver the therapy to an acceptable level of treatment integrity.

### Learning points

At the outset of the study there was concern whether the complex consent process would delay children accessing therapy, and a number of steps were built into the study protocol to ensure this was not the case, such as beginning to make contact with the local authority to explore issues of consent, whilst the clinical referral was being processed. Encouragingly these steps were largely successful. While contacting social workers was a challenge at times, it did not delay children accessing therapy, because the preliminary consent was obtained while the therapists were arranging the first consultation meeting. The need to seek consent from a number of different parties (the local authority, foster carer, the child and, depending on the child’s legal status, the birth parents) was also managed in such a way as not to delay treatment, or to reduce the proportion of eligible participants who agreed to take part. Likewise, despite concerns about the impact of placement moves on the capacity to collect complete data, all children who moved placement during the course of the study were successfully followed-up, with additional consent successfully obtained from new foster carers.

As shown in Fig. 1, a large proportion of looked after children referred to the Targeted CAMHS team were not eligible for the present study. In most cases this was because the initial team assessment suggested help could be more appropriately offered by a different service. Despite this, recruitment into the present study was acceptable due to the rate of recruitment of eligible families being much higher than expected. However some revision to the inclusion and exclusion criteria should be considered if conducting a definitive trial, such as extending the upper limit of the age range from 16 to 18, which was proposed by a number of foster carers and CAMHS therapists. A significant number of children in care are also under the age of 5, so future studies could also consider including these children and their carers; however feedback from the focus groups suggested greater modifications of the MBT model would be needed for this age group. Adaptations of MBT for this younger age group already exist, so it may be more appropriate to focus on adapting such interventions for use with children in care under the age of 5.

Retention of participants in the study was very high, and even when children moved placement during the course of the study, it was shown to be feasible to collect data from any new foster carers, who were also able to support the child in continuing with the therapeutic intervention, when this was considered to still be needed. However, a key issue that was identified concerned the timing of assessments. As the Service was set up to deliver short-term interventions (6–12 sessions), and have short waiting-times for those referred, follow-up assessments were planned for 12- and 24-weeks post-randomisation. However contrary to what was expected, young people had on average only attended three sessions by week 12, and often had not completed treatment by the final follow-up assessment at week 24, resulting in no outcome assessment following the end of treatment. Exploration of this pattern of attendance indicated that practical reasons meant that sessions were usually far more spaced out than had been anticipated, with 6–12 sessions being offered in most cases over a period longer than 6 months. This has obvious implications for the design of a future trial, as the current design may have missed changes related to treatment that occurred beyond the end of the research. For a future trial the design will need to ensure that the main outcome is timed following completion of treatment.

When considering whether the SDQ is the most appropriate screening and primary outcome measure for a study of this sort, a number of questions have been raised. A separate analysis of referral data in relation to clinical decision making suggests that the single-informant SDQ may not be an appropriate screening tool for the wellbeing of looked after children [27]. The self-report version of the SDQ is not validated for children below the age of 11, meaning that it could not be used as a main outcome measure in any future trial covering this wide age range (5–16). Moreover, the SDQ lacks questionnaire items which cover issues relevant to children in foster care, such as attachment, developmental trauma and relationships. If the SDQ cannot successfully identify children in foster care who are in need of support, it is also likely to be problematic as an outcome measure in a trial of this nature. Further work is needed to identify suitable primary outcome measures for research attempting to evaluate the impact of psychological interventions on the mental health and emotional well-being of children and young people in foster care.

## Conclusion

The study data indicates that it is feasible to carry out a randomised controlled trial evaluating the effectiveness of a family-format model of MBT for children in foster care in the context of targeted child and adolescent mental health services. Delivery of both the therapy and the research is practicable. Some adjustments would be required, especially planning of assessments to ensure that they map onto a realistic time-frame for treatment completion.

Given the recognised challenges in recruiting and carrying out clinical trials with this population, the findings of this study are highly encouraging. A full-scale definitive trial with follow-up at the end of all treatments is needed to determine efficacy. The positive findings in relation to feasibility and acceptability will be useful to other research groups who are planning clinical trials of psychological therapies to support the wellbeing of children in foster care.

## Additional file

Additional file 1: Results of additional measures. (ODT 12 kb)

## Data Availability

All anonymised data can be made available from the research team upon request.

## Abbreviations

CAMHS: Child and Adolescent Mental Health Service
CASUS: Child and Adolescent Service Use Schedule
CBT: Cognitive behavioural therapy
CHI-ESQ: Experience of service use schedule
CHU9D: Child Health Utility
CI: Chief investigator
CLA: Children looked after
CRN: Clinical Research Network
CSO: Clinical studies officer
CTSN: Clinical trials support network
CTU: Clinical trials unit
FCO: Full care order
GBOM: Goal-based outcome measure
LA: Local authority
MBT: Mentalization-based treatment
MRC: Medical research council
NIHR: National Institute of Health Research
PIRG: Public involvement in research group
PPI: Patient and public involvement
RA: Research assistant
RCADS: Revised child anxiety and depression scale
RCT: Randomised controlled trial
RfPB: Research for Patient Benefit
SDQ: Strengths and Difficulties Questionnaire
UCC: Usual Clinical Care
