# Supplementary material for: The Herts and Minds study: feasibility of a randomised controlled trial of Mentalization-Based Treatment versus usual care to support the wellbeing of children in foster care
Source: BMC Psychiatry. 2019 Jul 10;19:215. doi: 10.1186/s12888-019-2196-2 (PMC6617562; doi:10.1186/s12888-019-2196-2)
Supplement: Supplementary file 1 [file 12888_2019_2196_MOESM1_ESM.odt]

|  | Baseline | 12 Weeks | 24 Weeks |
| --- | --- | --- | --- |
|  | MBT Mean (SD) | UCC Mean (SD) | MBT Mean (SD) | UCC Mean (SD) | MBT Mean (SD) | UCC Mean (SD) |
| Five Minute Speech Sample (carer) - Reflective Functioning Scale [25] ~ |
|  |
| Total Score | 4.0 (1.0) | 5.1 (1.4) | 4.7 (1.1) | 4.6 (1.2) | 4.5 (1.3) | 4.7 (1.6) |
|  |  |  |  |  |  |  |
| Parent Stress Index – Short Form [28] † |
|  |
| Total Score | 141.0 (18.3) | 149.5 (17.6) | 139.3 (26.3) | 147.5 (18.1) | 138.0 (29.4) | 156.9 (18.4) |
| Parental Distress | 47.0 (6.1) | 49.8 (5.9) | 46.4 (8.8) | 49.2 (6.0) | 46.0 (9.8) | 52.3 (6.1) |
|  |  |  |  |  |  |  |
| Dysfunctional interaction | 47.0. (6.1) | 40.8 (6.4) | 46.4 (8.8) | 41.9 (5.5) | 46.0 (9.8) | 46. (6.7) |
|  |  |  |  |  |  |  |
| Difficult Child | 47.0 (6.1) | 37.7 (5.9) | 46.4 (8.8) | 39.7 (6.8) | 46.0 (9.8) | 42.1 (9.7) |
|  |  |  |  |  |  |  |
| Brief Parental Self-Efficacy Scale [29] ¥ |
|  |
| Total Score | 28.9 (2.6) | 28.8 (2.0) | 27.7 (2.9) | 28.6 (2.1) | 28.0 (2.2) | 28.6 (3.2) |
|  |  |  |  |  |  |  |
| Parenting Scale [30] ⱡ |
|  |
| Total Score | 2.8 (0.6) | 2.6 (0.5) | 2.8 (0.6) | 2.4 (0.5) | 2.8 (0.5) | 2.4 (0.5) |
| Laxness | 2.6 (0.8) | 2.4 (07) | 2.5 (0.9) | 2.1 (0.9) | 2.3 (0.6) | 2.3 (0.9) |
| Over reactivity | 2.3 (1.0) | 2.2 (1.0) | 2.6 (0.8) | 2.0 (0.8) | 2.6 (0.8) | 1.8 (0.6) |
| Hostility | 1.2 (0.3) | 1.1 (0.2) | 1.3 (0.5) | 1.1 (0.2) | 1.5 (0.7) | 1.2 (0.5) |
| Goal Based Outcome Measure [31] * |
|  |
| Foster Carer | - | - | 6.2 (1.5) | 4.0 (2.6) | 6.1 (3.0) | 4.3 (2.7) |
| Young Person | - | - | 3.3 (5.8) | 5.2(2.8) | 6.6 (2.3) | 3.4 (2.6) |
| Revised Child Anxiety and Depression Scale (RCADS) [32] † |
|  |
| Foster Carer-reported | 34.0 (20.0) | 34.7 (17.7) | 31.6 (16.6) | 26.3 (10.4) | 33.5 (19.9) | 24.6 (17.2) |
|  |  |  |  |  |  |  |
| Young Person -reported | 44.6 (31.1) | 31.2 (21.5) | 27.9 (23.3) | 23.6 (17.3) | 29.4 (24.2) | 22.8 (19.9) |
|  |  |  |  |  |  |  |
| Brief Assessment Checklist [33] † |
|  |
| Total Score | 19.8 (7.5) | 20.1 (6.6) | 18.2 (7.2) | 18.3 (5.9) | 16.7 (8.9) | 16.0 (7.2) |
|  |  |  |  |  |  |  |

Note: MBT is mentalization-therapy; UCC is usual clinical care; ~ A low score indicates a higher level of reflective functioning † high score means higher level of difficulties; ~ A low score indicates a higher level of reflective functioning; ⱡ high score represents a higher level of parental dysfunction; * high score indicates closer to reaching goals;  - measure was not assessed at the given time point
